# 

**Published:** 1852-11

**Authors:** 


### CONTENTS

OF THE

### MEDICAL EXAMINER.

NEW SERIES—NO. XCV.—NOVEMBER, 1852.

### ORIGINAL COMMUNICATIONS.

Adhesive Plaster as a means of making Extension in the Compound and other Fractures of the Lower Extremity. By S. D. Gross, M. D., Professor of Surgery in the University of Louisville - 685

Case of Difficult Labor from Deformity of the Fœtus. By H. B. Wilson, M. D., of Keedysville, Md. - - - 687

Mortality of Philadelphia for July, August and September, 1852, arranged from the Record kept at the Health Office. By Wilson Jewell, M. D. - - - - - - - 689

Experimental Researches applied to Physiology and Pathology. By E. Brown-Séquard, M. D., of Paris.

On the Mode of Action of some of the most active Poisons on the Nervous System - - - 698

On the Crossed Transmission of Impressions in the Spinal Cord - - - - - - - 703

### CLINICAL REPORTS.

Pennsylvania College, Ninth below Locust street. Service of Professor Gilbert. Reported by W. H. Gobrecht, M. D. - - 709

### BIBLIOGRAPHICAL NOTICES.

On Syphilis, Constitutional and Hereditary, and on Syphilitic Eruptions. By Erasmus Wilson, F. R. S., Author of "A Treatise on Diseases of the Skin," &c. With four colored Plates. - 723
Transactions of the Twenty-ninth Annual Meeting of the Medical Society of Virginia, together with the President's Annual Address and the Constitution of the Society - - - 730
The History, Diagnosis and Treatment of the Fevers of the United States. By Elisha Bartlett, M. D., Professor of Materia Medica and Medical Jurisprudence in the College of Physicians and Surgeons of the University of the State of New York, Member of the American Academy of Arts and Sciences, Author of an Essay on the Philosophy of Medical Science, &c. &c. - - - - - - - 731
Clinical Reports on Continued Fever, based on Analyses of one hundred and sixty-four cases, with remarks on the Management of Continued Fever, the identity of Typhus and Typhoid Fever, Diagnosis, &c. To which is added a Memoir on the Transportation and Diffusion by Contagion, of Typhoid Fever, as exemplified in the occurrence of the Disease at North Boston, Erie Co., N. Y. By Austin Flint, M. D., Professor of the Principles and Practice of Medicine, and of Clinical Medicine, in the University of Buffalo, and Editor of the Buffalo Medical Journal - - - - - - 731
Curiosities of the Microscope, or Illustrations of the minute parts of Creation, adapted to the capacity of the young, with colored illustrations. By Rev. J. A. Wythes, M. D. - - - 733

### EDITORIAL.

The Medical School of Philadelphia - - - - 733
The Medical School of London - - - - - 734
University of London - - - - - - 734
Royal College of Physicians, London - - - - 738
Royal College of Surgeons of England - - - - 739
St. Bartholomew's Hospital - - - - - - 742
Charing-Cross Hospital - - - - - - 742
St. George's Hospital - - - - - - 743

Guy's Hospital - - - - - - - 743
King's College Hospital - - - - - - 743
The London Hospital - - - - - - 744
St. Mary's Hospital - - - - - - - 744
Middlesex Hospital - - - - - - - 744
Royal Free Hospital - - - - - - 744
University College Hospital - - - - - - 745
Westminster Hospital - - - - - - 745
School of Anatomy and Medicine adjoining St. George's Hospital - 746
Hunterian School of Medicine - - - - - 746
Errata - - - - - - - - 746

MEDICAL NEWS.

Tribute to Dr. Brown-Séquard - - - - - 747
Tribute to Dr. Hooker - - - - - - 747
Appointment - - - - - - - 748
Emigrant's Hospital, Ward's Island, N. Y. - - - - 748
New Medical Schools - - - - - - 748
Professorial Appointments - - - - - - 748
Cholera - - - - - - - . 749
Baron Humboldt - - - - - - . 749
Medical Fees in Spain - - - - - - 749
M. Wurtz's Alcool entylique - - - - - 749
Death of M. Dize - - - - - - - 749
Autobiography of Berzelius - - - - - 750

### RECORD OF MEDICAL SCIENCE.

MATERIA MEDICA AND THERAPEUTICS.

On the use of Manganese as an Adjuvant to Iron - - - 750

